# Medicine and the media: Medical experts’ problems and solutions while working with journalists

**DOI:** 10.1371/journal.pone.0220897

**Published:** 2019-09-12

**Authors:** Anna Larsson, Susanna Appel, Carl Johan Sundberg, Mårten Rosenqvist

**Affiliations:** 1 Karolinska Institutet, Department of Clinical Science, Danderyd University Hospital, Danderyd, Sweden; 2 KTH Royal Institute of Technology, Science for Life Laboratory, Solna, Sweden; 3 Department of Physiology and Pharmacology, Karolinska Institutet, Stockholm, Sweden; 4 Department of Learning, Informatics, Management and Ethics, Karolinska Institutet, Stockholm, Sweden; Universidade de Brasilia, BRAZIL

## Abstract

Medical experts are one of the main sources used by journalists in reporting on medical science. This study aims to 1) identify problems that medical experts encounter in contacts with the media representatives, 2) elucidate their attitudes about interactions with journalists and 3) reflect on solutions that could improve the quality of medical journalism.

By using in-depth interviews, focus groups and a survey directed to 600 medical experts in 21 countries, this cohort study elucidates medical experts’ experiences and views on participating in popular media. A strong interest in interacting with the media was identified among the experts, where nearly one fifth of the respondents in the survey claimed that they contacted the media more than 10 times per year. Six obstacles for improving the quality of medical reporting in the media were found: deadlines, headlines, choice of topic or angle, journalist’s level of medical knowledge, differences in professional culture and colleagues’ opinions.

The main concern among experts was that short deadlines and exaggerated headlines could harm journalistic quality. It is possible that this is partly due to ongoing changes in the media landscape with many new platforms and less control functions. Nevertheless, for several reasons many experts have great interest in interacting with the media, something that could contribute to better communication and fewer misunderstandings.

Our results highlight factors like expert networks, media training for scientists and regular meetings that may facilitate communication between medical experts and medical reporters.

## Introduction

During the past few decades, the mass media has emerged as an important and effective channel for disseminating medical news, not only to the lay public but also to health professionals and policymakers. Medical messages in the media influence health-related decisions made by lay people as well as by health professionals and policy makers. Medical experts are among the most trusted sources for journalists reporting on medical news. Their contribution is essential to a high-quality medical journalism.

Previous studies have shown that when making health-related decisions, lay people as well as health professionals and policy makers rely on medical information distributed by the media [1–5].

To empower patients and expand health literacy, journalists and experts need to have effective interactions centered on relevant and reliable medical news [6].

When pandemics or any other type of emergent health crises threaten society, the importance of lay people’s trust in medical science is crucial, as is their confidence in reliable medical services. Considering the outsized role the media has in disseminating medical news it is extremely important that the medical material they distribute is accurate and relevant [7–8].

The new media environment, including the Internet and all social media content, along with new technologies for dispersing information and opinion have considerably changed the working conditions in newsrooms. Few studies have hitherto attempted to elucidate the process that develops scientific findings into journalistic pieces in today’s media landscape. With increasing opportunities for several actors to publish more often and on different media platforms, the frequency of and the time to deadlines for news reporters have changed dramatically. The time allotted to find news, research facts, check details, write stories and make layout has shortened, and fewer sources are used [8–10].

In the quickly changing media landscape where anybody can forward medical messages to a large audience the impact of reliable sources, *e*.*g*. independent physicians, with updated knowledge cannot be overestimated [3–4].

Over the past few decades, several studies have identified differences in values and goals between the expectations of experts and journalists [5, 11]. Representatives from the medical community have accused some journalism of being alarmistic, commercially influenced or misleading [11–14]. At the other end of the spectrum, journalists have reported lack of production time, available text space or broadcasting duration as well as knowledge as barriers producing high quality work, as well as difficulties finding experts who are independent and willing to assist the media [6]. To improve medical journalism it has been proposed that a greater understanding of working conditions in the media is needed [4].

Previous studies have shown that the medical expert and journalist professional cultures have been approaching each other and that media researchers have begun to recognize the symbiotic character of media-science interactions [8, 15, 16].

The aims of this study were to 1) identify problems that medical experts encounter in contacts with the media representatives, 2) elucidate their attitudes about interactions with journalists and 3) reflect on solutions that could improve the quality of medical journalism.

## Material and methods

### Interviews

Two in depth interviews were conducted with medical experts who were chosen because of their extensive experience working with journalists. The main aim was to identify recurrent problem areas and suggested solutions. The interviews were taped, transcribed and used in the construction of a survey.

### Focus groups

We organized two focus groups with 14 participants (eight respective six) from different countries. The experts in the first group were identified by their active participation in media activities within the annual congress of the European Society of Cardiology. The inclusion criteria were: being cardiologists and researchers, having presented scientific material at two or more press conferences, and having followed up on the media coverage afterwards. The participants in the second group were chosen by their regular appearance in the Swedish media as sources for information about antibiotics and microbial resistance. Inclusion criteria stated that participants should be full time medical scientists and/or physicians with experience of speaking with the media. Beside they should have been interviewed as medical experts in Swedish mass media at least three times during the previous six months.

The focus group leader (AL) posed open questions and allowed a free discussion among the participants. The questions followed a prepared list with several possible obstacles and strategies for addressing them.

### Survey

Based on the data extracted from the focus groups and the in-depth interviews, an attitude survey instrument was constructed. It consisted of 21 closed questions together with one open field, allowing respondents to comment freely *(S Appendix*). The questions were validated by a pilot distribution of the survey to ten Swedish medical experts with media experience. The survey was set up as an invitation only internet-based questionnaire. Six hundred persons from 21 countries across Europe, North America and Australia were selected to be invited as study subjects. The inclusion criterion was that the person must have previously appeared as a medical expert (source linked to specific report or interview person) in a daily media outlet with one or more pages designated to medical news that were also published on their website. From a sample of the largest (measured in circulation) daily media with an edition in English, French or Swedish distributed in Europe, USA/Canada and Australia, we chose those with an online page designated on medicine. 17 media outlets were identified. From their medical pages online we identified experts who were quoted or interviewed in articles about medical issues during one year (2009).

All selected individuals were sent an e-mail invitation to participate in the survey. Any invited person who did not submit answers to the questionnaire was sent up to three reminders. To ensure anonymity, we employed a computerized automatic system for all re-distributions of the survey as well as for subsequent data collection and processing.

As this study design is not comprised by Swedish ethical law, no information regarding personal integrity was used, all data were anonymized and no patients were included, no ethical application was submitted.

## Results

### Focus groups

The participants in the focus groups were told to speak freely about their experiences in the area and to express their views on problems that they encountered in their relations with the media.

The headlines were of most concern to the members in both groups.

*“The headline maker does not have the time to read this work very well and may make a wrong headline, which would ruin the whole interview, and that happens quite often*.”

*“The shorter items in the media and the space you have, the worse it gets. The worst case is headline news. Physicians should be very weary of discussing medical topics when space is too constrained*.”

*“One may have full control of what you’re saying yourself and what appears in the newspaper–except for the headlines. The headlines are very dangerous because they are made in the last second*.”

Members in the group of infection experts described the problems with media’s short production times, which can threaten the quality of the journalistic items.

*“…most unpleasant is when the hurry leads to short items that are supposed to be black or white, and you have to be very clear about which worries the public should have, and what it means to a single patient. You have to develop the context, but there is no place for that*.”

*“…when dealing with an inexperienced journalist I often ask to get to read the manuscript before publication, and they have hitherto agreed, but sometimes it has been close to deadline and difficult to manage*.”

Exaggeration of less important facts and biased selection of less important topics were also highlighted.

*“One day they tell you that coffee is good, the next that it is bad, or that white egg is good, next time is yellow egg good, whatever. There is important news on prevention, lifestyle, and drugs which gets buried into this noise, this background noise of random news*.”

Different roles and aims for journalists and experts were discussed in both focus groups, as was different education levels of media representatives.

*“There is a different aim in the professions. The aim of journalists is to get news, it does not matter if it is real or if it is true. And the aim of the doctor is to bring reliable news which is often much later than the journalist wants to have it*.”

*“If the journalist has a low level of knowledge, the message could easily be quite different from what you intended, and that could be disastrous, if you are the spokesperson for an organization for example*.”

*“When I make a statement to the press, those who will criticize me are not the journalists or the readers. It is my colleagues, they will scrutinize me, and if they find anything that they do not agree with, they will give me a tough time*.”

Some of the experts reported that they had personal strategies while being interviewed. Some demanded to approve their quotations before publishing, some asked to get the questions in advance, and one expert admitted to having kept some information from journalist fearing that the media would misuse it, perhaps hurting vulnerable patients. Most experts also admitted having used media contacts to relay their own messages.

*“You have to set up your own agenda and know what message you need to get across, regardless which questions you get”*.

*“One selling point is, which is very efficient but not satisfying, is that you sell the interesting news to the lay media, and the scientific writers will wake up because they see everybody is interested in that and then the thing comes into motion. It's dangerous in a way but that's very successful*.”

### Survey

A total of 118 experts, equalling a response rate of 22%, answered the survey and were characterized by their extensive experience in the field of medicine (*Tables 1 and 2).*

**Table 1:** Characteristics of medical expert informants’ occupation.

Occupation	Proportion %
Academic research	74
Health care	31
Public authority	17
Charit organization	8
Private company	6
Other	4

**Table 2:** Characteristics of medical expert informants’ experience.

Medical experience	Proportion %
1–5 years	5
6–15 years	17
16–25 years	29
More than 25years	50

The respondents were based in 13 different countries and most of them were active in academic research. Of the respondents, 38% was from USA, 26% from Sweden, 14% from UK and almost 9% from Australia. We suggest that this is due to a more active discussion of the issues among experts and journalists in these countries. Since the media situation differs among countries, we would not make any consideration about our study having a larger impact than the numbers show.

Almost 50% had worked for more than 25 years. Close to two thirds were men and the majority resided in Europe or North America (*Table 3*).

**Table 3:** Medical informants’ main country of residence.

Country	Respondents(number)
USA	50
Sweden	32
U K	18
Australia	12
France	5
Denmark	3
Japan	1
Holland NewZeeland Spain	1 1 1

The survey showed that the respondents had extensive experience with the media, and a majority had more than ten contacts with journalists annually. 74% of the experts from USA had more than six contacts with journalists each year, compared to medical specialists from Europe where 58% had more than six media contacts yearly. Usually the expert was contacted by a journalist, but more than half of the respondents had themselves contacted the media for different reasons during the past few years. Almost one-fifth of respondents previously used to contact the media themselves more than ten times a year. Approximately half of the respondents reported that their aim was to spread information about either their own medical field or a specific study, but correction of facts in material published by the media was also a common purpose. More than 40% of respondents who contacted the media wanted to create debate or form opinion. The nature of media contacts is shown in Tables 4 and 5. (*Tables 4 and 5).*

**Table 4:** Frequency of media contacts.

Frequency of contacts	Proportion %
Never	1
1–3 times/year	19
4–6 times/year	15
7–9 times/year	14
10 times/year or more	51

**Table 5:** Intentions of media contacts.

Intentions of initiated media contacts	Proportion %
Spread information on own medical field	50
Spread information on study/other subject	54
Correct facts in already published news	49
Other corrections	25
Debate or form opinion	42
Create publicity for own research group	25

**Fig 1:**
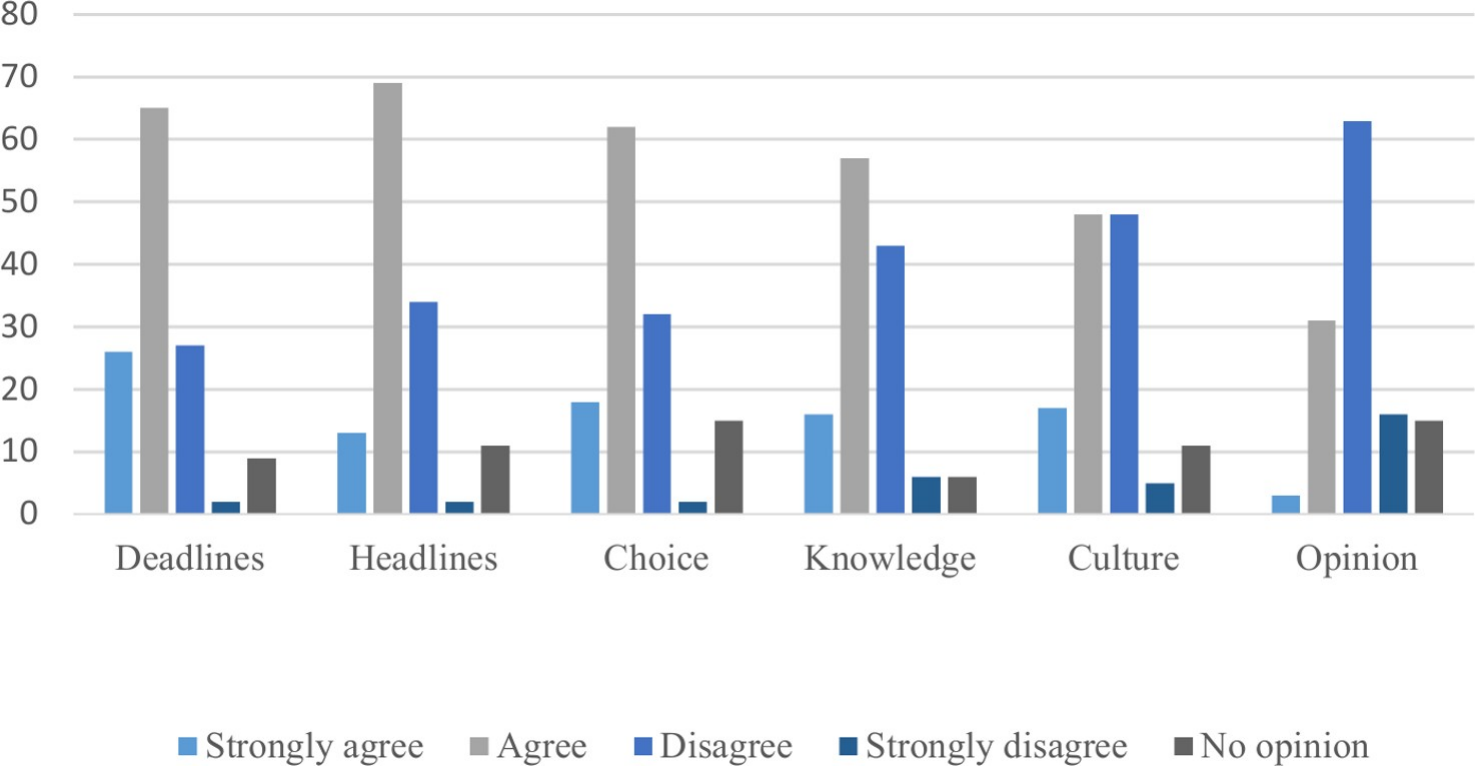
Experts’ assessments of problems in media contacts.

Among the identified problems were problems concerning headlines and deadlines. *(Fig 1).*

A majority stated that the phrasing of headlines and too short deadlines were barriers to a good outcome of meeting with the media. Media’s choice of topics was a main concern to a majority of the respondents, as was journalists’ incomplete basic medical knowledge. More experts in UK and Australia agreed that lacking medical knowledge was a barrier—61% respectively 68%—compared to Sweden and USA where 50% and 52% thought that journalists’ incomplete knowledge was an obstacle.

A majority of experts stated that colleague’s opinions were not an obstacle in interaction with journalists.

**Fig 2:**
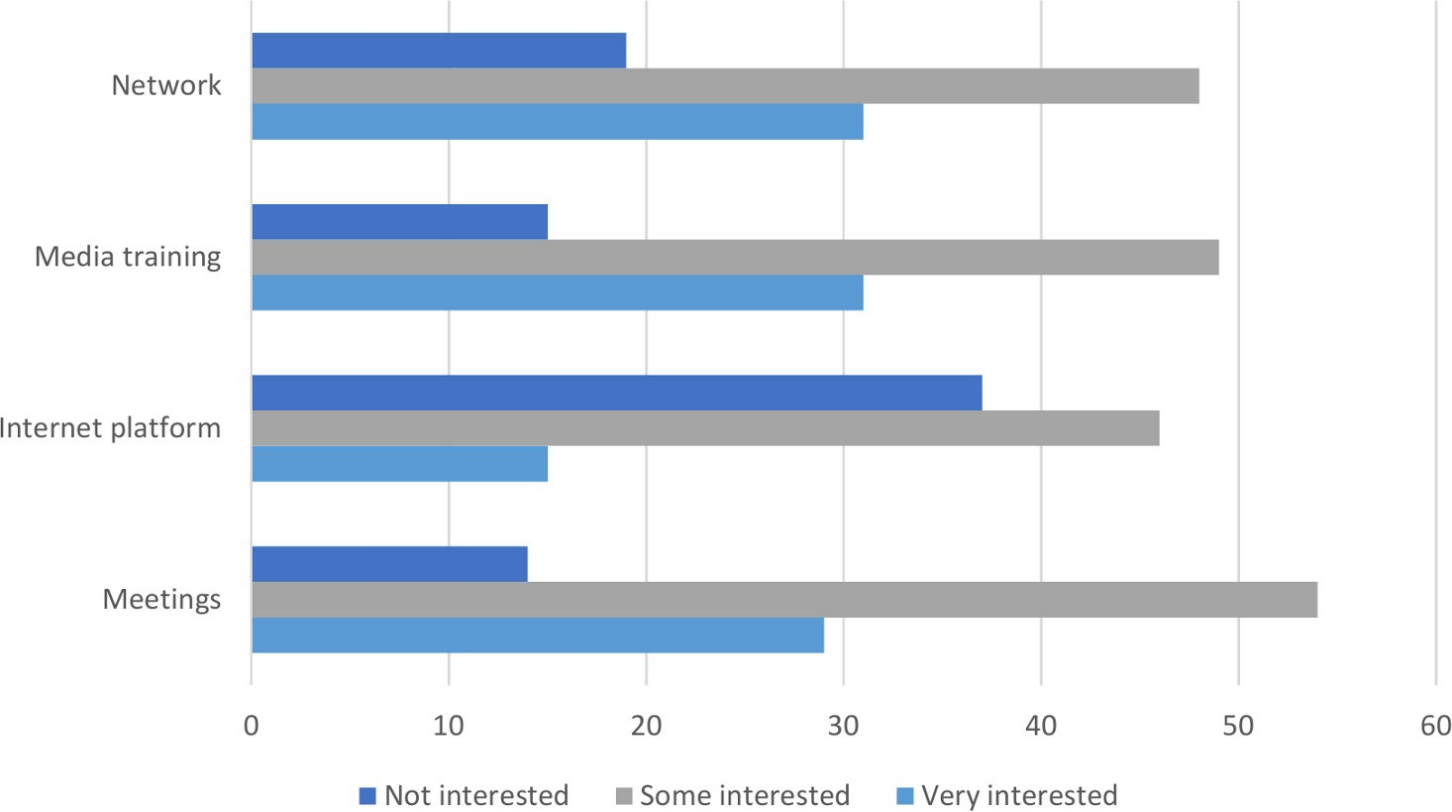
Experts’ interest in suggested solutions.

The respondents were asked about different strategies that could improve the interaction between journalists and scientists. Improvement of journalists’ basic medical knowledge was suggested as a means to get a better medical coverage in the lay media. This could be achieved through regular meetings between experts and reporters in which important new findings could be discussed and explained. A network available for journalists wanting expert opinions to validate news stories before publishing was approved as an improving measure for medical reporting. The respondents were also in favor of more media training for experts to understand the preconditions in the media market. *(Fig 2).*

## Discussion

A significant finding from this study is the strong interest of scientists to interact with media representatives. Many of the respondents chose to contact media themselves. Nearly 20% report that they had contacted the media more than ten times a year, the main aim being either to shed light on their own research field, to create debate or to shape opinion. This could be a reflection of current changes in the ways people communicate in the digital era, with more possibilities to elucidate own opinions on different platforms. This is certainly interesting as both a field for further studies as well as a medium to investigate how these changes affect the form and content of medical information.

According to previous studies, the growing interaction of medicine and the media has been a continuing trend since the 1980s. Due to the demand from society at large for more science-based health information, the scientific community has incrementally abandoned its earlier hesitance to communicate with groupings outside academia [17]. A survey of the literature shows that this is not only a result of individual initiatives. To interact with media is considered a part of the modern scientist’s role, and refusing contacts with journalists has become unacceptable [16]. Earlier studies have found that although experts are critical of media coverage in general, they still appreciate their own experience working with journalists [16, 18]. An increasing frequency of contacts between scientists and journalists could be a result. (*Table 4*). Our data shows that 74% of the respondents from USA had six or more media contacts per year, while the corresponding fraction for European experts was 58%.

Interactions between medicine and the media are viewed as important in promoting science literacy, but they are also viewed as important ways to advance one’s own career [19]. There are several factors that stimulate media interaction for scientists’–and universities’—growing interest using the media to promote their activities. This is reflected in the development of large information offices at medical universities and health care organizations. Visibility in the media is considered an effective tool to guarantee support from the public and from the politicians as well as increasing funding for their operation [20–21]. Previous studies show a consciousness among scientists about the importance of their presence in the media and that responding to journalists is a professional duty [16].

### Focus groups

Our findings show that several barriers still make contacts challenging between medicine and the media. According to our results, the very short deadlines in newsrooms and exaggerated headlines are perceived as important risk factors for low-quality medical journalism. Several studies have pointed out that the ongoing restructuring of newspapers have brought staff cuts, resulting in increased time constraints and work overload experienced by few remaining employed journalists [22, 23, 24]. Most likely, this strongly contributes to a diminishing quality of science reporting [9]. Another time-consuming factor is the 24–7 news cycle that demands continuous rapid updates of an ongoing story [8]. To support accurate, valid and relevant medical reporting in the lay media, experts at universities and health care organizations need to better understand the working conditions for journalists and facilitate their access to resources and technical expertise [4].

Our focus discussions showed that experts sometimes disapprove of the media’s preference for popular, often overly simplified topics or angles with less relevance for health rather than exploring more complex and impactful areas. This phenomenon could be a result of a dependence on the need to maximize reader-, viewer- and listenership, which is measured in the number of, downloads and clicks. Another reason could be the difficulties journalists have in finding independent researchers and accessing resources and technical support from the medical community—a weakness easily capable of compromising the result of their work. This is for example reflected in a previous study of how the coverage of medications in the lay media leaves out risks, costs and financial ties between study groups and pharmaceutical manufacturers [14].

Since the Internet has transformed the communication systems globally, some of the problems of the interaction between medicine and the media have grown, for example short deadlines. Before this era there is evidence that other barriers between the two professions existed. An analysis by Nelkin [17] shows that scientists had difficulty understanding the role of journalists as watchdogs and the importance of a free press in a democratic society. Another study from 1992 describes the views of scientists who presented their studies in New England Journal of Medicine and JAMA. When asked of their impression of media coverage, they concluded that the most positive factors were that it informed the scientific community and gave the public possibility to understand new findings. It also improved the image of the profession. The most negative was that it took too much time, gave the impression of the researcher seeking publicity and that it created jealousy among colleagues [25].

### Survey

The lack of medical knowledge at news desks has been brought up by several of our respondents. To have to explain scientific and medical facts for journalists with limited experience and no medical education is seen as a significant barrier to effective interaction with the media. Experts point out that this is time consuming and could lead to misunderstandings. Earlier studies have shown that journalists with low medical knowledge are a risk factor for low quality coverage of important medical findings. Exaggerations or omission of important facts could be an effect and has been observed in the discussion about vaccination, measles and autism [26–27]. Our data show different views on journalists lacking medical knowledge. More experts in UK and Australia agrees with this as a barrier (61% and 68% respectively) compared to Sweden and USA (50% and 52% respectively). Overly frequent media contact has previously been seen as a risk to a trustworthy professional scientific reputation for scientists. The change of norms in this area has been studied and the diminished impact of colleagues’ opinions on media participation shown in our results is reflected in studies of the change of views on the science-media relationship [28].

The respondents were asked about different strategies that could ameliorate the relationship between journalists and scientists. Improvement of journalists’ basic medical knowledge was suggested as a means to producing better coverage of medical issues in the lay media. This could be achieved through regular meetings between experts and reporters in which important new findings could be discussed and explained. A network available to journalists who want expert opinions that can help them validate news stories before publishing was lauded as a potential improvement to medical reporting. A previous study on the need of journalists working with medical stories show similar suggestions. A great majority wanted access to experts in various areas in health and medicine. Many of them were also interested in learning strategies to prepare more entertaining and factual pieces, both entertaining and ‘salable’ [6]. The expert respondents were also in favor of more media training for medical experts to help them understand the conditions journalists are subject to in the media marketplace and landscape (*Fig 2*).

## Limitations

The media landscape has gone through important changes during the last 20 years. Any study conducted in this period would be affected by the large modifications that have occurred. A lot of possibilities for individuals and new groups to publish material, *e*.*g*. in social media, have emerged and made more medical information accessible to everyone. This could have changed medical experts’ views about headlines and deadlines, overall quality of medical content in the media and the best ways of spreading medical information to the public. The low response rate, 22%, could have led to a selection of participating medical experts who were interested in media and communicating with the public, whereas the majority who did not respond might have arrived at other responses to our questions. One study has shown that specialists with leadership functions and research productivity interact more frequently with the media than do junior researchers [29], which also could have had an impact on the outcome.

## Conclusions

There were considerable worries among medical experts about short deadlines and unreliable headlines that jeopardize the medical messages in the media.

This implies that high-quality medical journalism could be at risk in todays’ changing media environment. The risk is not only that lay people will get medical information of low quality, but also that the publics’ confidence in science and medicine will erode. Some of the reasons for this are found in shorter production times in news departments, fewer specialized reporters, badly phrased headlines and experts willing to comment on stories in spite of conflict of interest.

Our results show a strong interest from scientists to interact with media representatives with the purpose of spreading medical information and correct facts in already published news stories.

The medical expert informants propose more frequent contacts between experts and journalists, regular meetings and a network of experts for reporters in need of corroborating experts about medical news. In addition, training for medical experts about working conditions for the media could further enhance the understanding of the two professional cultures.
